# New Therapeutic Paradigms and Guidelines in the Management of Pulmonary Arterial Hypertension

**DOI:** 10.18553/jmcp.2016.22.3-a.s3

**Published:** 2016-03

**Authors:** Nicholas S. Hill, Michael J. Cawley, Cherilyn L. Heggen-Peay

## Abstract

**BACKGROUND:**

Recent and ongoing developments in the diagnosis, treatment, and management of pulmonary arterial hypertension (PAH) provide deeper insights into pathogenic mechanisms. Approvals of new pharmacotherapies that improve function and reduce morbidity and mortality risks; advances in clinical trial methods, including long-term, event-driven studies with clinically relevant and patient-centered endpoints; and trial results support a new therapeutic management strategy. This new paradigm involves initial treatment with combined therapies that act through different disease pathways. In addition, 2 new sets of clinical practice guidelines for PAH have been published since June 2014. Despite these advances, major gaps have been documented in the diagnosis, treatment, and management of patients with PAH.

**OBJECTIVE:**

To present current knowledge and evidence on PAH to support managed care professionals and providers in achieving accurate differential diagnosis, promptly referring patients to specialists as necessary, and ensuring that patients receive appropriate, guideline-directed therapies.

**SUMMARY:**

Major gaps in the quality of care provided to patients with PAH include oversights in clinicians’ recognition of symptoms, delays in diagnosis, and misdiagnosis ensuing from incomplete evaluations, delays in referral of patients to centers of expertise and initiation of therapy, and inappropriate treatment regimens. To address deficiencies in PAH diagnosis, new practice guidelines emphasize the essential role of right heart catheterization in characterizing and confirming the disease, as well as referral to expert pulmonary hypertension centers to ensure appropriate evaluation and treatment. Updated disease and functional classifications of PAH, along with new research findings on prognostic factors and effects of comorbid conditions, offer key support for making effective therapy and management decisions for patients with PAH at different risk levels and stages of the disease. Since 2013, the U.S. Food and Drug Administration has approved new PAH therapies in the classes of endothelin receptor antagonists, guanylate cyclase stimulators, prostacyclin analogues, and prostacyclin receptor agonists. As demonstrated through phase 3 clinical trials, these generally well-tolerated therapies delay disease progression, improve hemodynamic and functional status, and decrease numbers of hospitalizations. Moreover, 2 sets of recently published guidelines—developed by the American College of Chest Physicians and the European Society of Cardiology/European Respiratory Society—provide evidence-based and expert consensus recommendations for achieving PAH treatment goals. The most recent guidelines include a recommendation for upfront combination therapy for patients with moderate disease, which is supported by new comparative clinical trial evidence. As addressed in this article, these advances in the field of PAH have important implications for managed care and clinical practice, including considerations of cost-benefit outcomes associated with different management strategies.

Pulmonary arterial hypertension (PAH) is a rare but complex chronic disease of the pulmonary vasculature that leads to right ventricular failure and eventually death if left untreated. PAH is a type of pulmonary hypertension (PH), which generally refers to high blood pressure in arteries of the lungs and may be caused by a variety of conditions.

As an incurable progressive disease with a poor prognosis, especially for patients who do not receive appropriate treatment, PAH has been the subject of intense research in recent decades. These efforts have resulted in major advances to improve diagnostic accuracy, support the development of targeted therapies, and develop a deeper understanding of optimal management strategies. However, a number of gaps in PAH management pose barriers to achieving treatment goals. Key documented gaps include oversights in clinicians’ recognition of symptoms, delays in diagnosis, and misdiagnosis ensuing from incomplete evaluations, delays in referral of patients to centers of expertise and initiation of therapy, and inappropriate treatment regimens.^1-4^ Moreover, the complex diagnostic process and management of the disease can result in high costs and utilization of health care resources, underscoring the importance of applying evidence-based and expert consensus guidelines to all aspects of patient care.

This article presents current knowledge and evidence on PAH to support managed care professionals and providers in achieving accurate differential diagnosis, promptly referring patients to specialists as necessary, and ensuring that patients receive appropriate, guideline-directed therapies.

## Epidemiology of PAH

Based on an analysis of the REVEAL Registry, the prevalence and incidence of PAH in the United States were 12.4 cases per million and 2.3 cases per million annually between 2006 and 2007.^5^ In contemporary European registries, prevalence and incidence have been estimated at approximately 6 to 15 cases per million and 1 to 2 cases per million annually.^6^ According to a landmark National Institutes of Health (NIH) study conducted in the 1980s, 5-year survival for patients with PAH was 37%.^6^ Although mortality rates appear to have improved in the last few decades, most likely due to many recent advances in the diagnosis and treatment of PAH, the disease remains progressive, carrying high morbidity and mortality. Analysis of data from the Pulmonary Hypertension Connection (PHC) registry, which evaluated patients between 1982 and 2006, showed that survival has improved over time.^7^ Both the PHC registry and REVEAL data estimate the current rate of 5-year survival of PAH patients to be about 60%, with rates generally lower for blacks (versus whites), men, and the elderly.^6,8^ Based on an analysis of the REVEAL Registry, PAH is shown to affect more women than men at a rate of 3.6 to 1, and the mean age at diagnosis is 47 years.^5^

## Challenges in Diagnosis and Treatment of PAH

A major challenge in the management of patients with PAH is ensuring early diagnosis. As reported in a REVEAL Registry analysis, the median time from PAH symptom onset to diagnosis using right heart catheterization (RHC) was 1.1 years, and 21.1% of patients had symptoms more than 2 years before diagnosis.^9^ In addition, this analysis identified several factors that are associated with greater likelihood of delayed or missed diagnosis, including younger age, presence of other respiratory conditions such as obstructive lung disease and sleep apnea, and less severely impaired right ventricular function at time of RHC. Other potential factors that contribute to delayed diagnosis may be that patients remain asymptomatic or present with nonspecific symptoms that lead to workup of other more common diseases that are more familiar to clinicians. Also, lack of access to health care may partially explain the delayed diagnosis, especially in younger populations.^1^ Diagnosis of PAH at a late stage is associated with a poor prognosis for survival, which highlights the need for increasing awareness and education among managed care professionals and clinicians. A key goal is to routinely consider PAH in differential diagnoses, especially in younger patients and those with other pulmonary diagnoses who have symptoms out of proportion to their disease or who are not responding to treatment.^1^

Another challenge in the management of patients with PAH is delayed initiation of appropriate treatment. Despite current practice guidelines that advocate for early referral of patients to expert centers, the RePHerral study detected significant delays. At the time of referral, 61% of the patients in this study were already in advanced stages of disease, and of these patients only 30% were on any PAH-specific medications.^2^

Even when PAH is recognized as a potential diagnosis, practice trends indicate significant gaps in care and underutilization of evidence-based guidelines. In a 2013 study, using data from the Pulmonary Arterial Hypertension Quality Enhancement Research Initiative (PAH-QuERI), investigators found that, before enrollment, only 6% (n = 791) of patients had undergone all tests recommended by the American College of Chest Physicians (ACCP). Despite an automated reminder program, at the 12-month follow-up only 7% of patients had completed all ACCP-recommended tests. RHC, which is required for a definitive PAH diagnosis, was performed in 90% of patients at enrollment; however, after 12 months of follow-up, only 2 additional RHCs were performed among the 77 patients who did not have the test initially.^3^

Observed treatment practice patterns also demonstrate that management guidelines are underutilized. The RePHerral study showed that of the patients who were on PAH-specific medications at referral, 57% of the prescribed medications were not consistent with published guideline recommendations.^2^ Significant disparities in treatment practices have been reported among clinicians even for clinical scenarios with clear, evidence-based management guidelines.^10^ For example, the 2014 CHEST guidelines from the ACCP, and the 2015 European Society of Cardiology and the European Respiratory Society (ESC/ERS) guidelines recommend that patients with severe disease receive infused prostacyclin analogues.^11,12^ Yet in the REVEAL Registry, only 56% of patients with a PAH-related death had been treated with intravenous prostacyclin analogue therapy.^4^ It is unknown why these patients with severe disease were not receiving guideline-directed prostacyclin analogue therapy. Possible explanations raised by the authors in the discussion included both physician- and patient-related barriers, such as the complicated delivery of prostacyclin analogue therapy, as well as potential insufficiencies in insurance coverage.

The guidelines also recommend that calcium channel blockers (CCBs) to treat PAH only be used in patients who demonstrate vasoreactivity on RHC.^11,12^ Nevertheless, in the PAH-QuERI analysis, only 7% of patients being treated with CCBs for PAH met guidelines for vasoreactivity.^3^ This is especially concerning because inappropriate use of CCBs can delay initiating other more effective PAH treatments and can also worsen right ventricular function in nonvasoreactive patients.^3^ These management inconsistencies and the complexity of PAH care underscore the need for continuing education for clinicians on PAH guidelines, including evidence-based therapies, the importance of diagnostic testing for accurate diagnosis and classification, and referral to PAH care centers. Program-level tools, such as standing orders, should also be considered to improve compliance with diagnostic and treatment recommendations. Furthermore, additional research is needed to better understand why clinicians do not consistently follow evidence-based and expert consensus recommendations.

## Cost Burden of PAH

Cost burden is another major managed care challenge in PAH. Although a relatively rare disease, PAH is associated with high costs and resource utilization to the U.S. health care system for both patients and payers. In recent analyses of large commercial claims databases, patients with PAH had significantly higher per-patient-per-month (PPPM) health care utilization compared with patients who did not have PAH. Higher utilization for PAH was reported across outpatient, inpatient, emergency room, and pharmacy services.^13^ Estimated direct PPPM costs for PAH patients are up to 4 to 5 times higher than for matched control patients with similar age, sex, geographic region, and employment status.^14^ The study also found that PAH patients had significantly higher baseline rates of chronic comorbidities such as hypertension, diabetes mellitus, and congestive heart failure compared with control group patients. In a recent study of 504 patients with PAH in a large U.S. managed health plan, average total PAH-related health care costs were estimated to be $9,723 PPPM for the baseline period and $8,187 PPPM in the 12-month follow-up period, translating to average annual costs per patient of $116,681 and $98,243, respectively.^15^ A cost breakdown analysis in this study demonstrated that the primary cost driver was inpatient care, accounting for nearly 70% of total direct costs in the baseline period.^15^

Compared with patients who have other primary diagnoses, PAH patients have a higher rate of comorbidities and incur significantly higher costs and longer lengths of hospital stay for primary conditions; moreover, patients with PAH are more likely to be readmitted after initial hospitalizations.^16,17^ In a retrospective database study, length of stay among commercially insured PAH patients with a PAH-related hospitalization was 14.2 days compared with 10.2 days in patients with other diagnoses.^18^ Among Medicare patients with PAH in the study, lengths of stay were 16.7 and 12.3 days, respectively. The investigators also reported that 50.6% of PAH patients hospitalized for a PAH-related diagnosis were readmitted within 1 year. Not surprisingly, this translated to a noteworthy difference in hospitalization costs for patients with a diagnosis of PAH compared with patients who had other diagnoses. Mean costs for the 2 groups were $61,922 versus $42,455, respectively.^18^

In another study, more than 75% of patients with PAH were readmitted to the hospital within 1 year of an initial PAH-related hospitalization, and of those who were readmitted, more than 20% were back in the hospital within 30 days of their initial discharge, resulting in a mean cumulative cost of $71,622 and mean length of stay of 24.5 days.^19^ Thus, the substantial health care costs associated with PAH highlight the need for improved managed care and clinical processes to promote more effective treatment outcomes and avoid preventable rehospitalizations.^17^

Pharmaceutical costs also contribute to overall medical costs in PAH. Recent data have demonstrated that of 8 agents approved by the U.S. Food and Drug Administration (FDA)—including CCBs, phosphodiesterase type 5 (PDE-5) inhibitors, endothelin receptor antagonists (ERAs), and prostacyclin analogues—the total annual per patient cost of treatment ranged from $18,000 to $244,000.^20^ A 2014 study examined health care costs for PAH patients before and 12 months after initiation of PAH-indicated medication. Although pharmacy costs increased significantly from the baseline to follow-up period ($6,440 to $38,514), they were offset by significantly lower medical costs in the follow-up period ($110,241 to $59,729), attributable to fewer outpatient visits and inpatient stays.^15^ The high pharmacy costs and the increasing number of available medications with varying administration routes, safety, and efficacy profiles complicate decision making and emphasize the need for additional cost-effectiveness analyses of the numerous treatment options for PAH.

## Clinical and Functional Classification, Treatment and Management Goals, and Prognostic Factors in PAH

Effective therapy and management decisions for patients with PAH depend on a comprehensive understanding of clinical and functional classifications (FCs) of the disease, established treatment and management goals, and factors that predict prognosis. These aspects of PAH are addressed in this section.

### Classification

Over the last few decades, better understanding of pathological findings, disease mechanisms, hemodynamic characteristics, and management of PAH has led to development of the current classification system, which separates PH into 5 groups. Group 1 PAH is distinguished by precapillary PH (mean pulmonary arterial pressure ≥ 25 mm Hg and pulmonary arterial wedge pressure (PAWP) ≤ 15 mm Hg) and a pulmonary vascular resistance > 3 Wood units (Table 1).^12,21,22^ Group 1 PAH includes 4 subgroups: idiopathic PAH (IPAH), heritable PAH (HPAH), drug/toxin-induced PAH, and associated PAH (APAH).^23^ IPAH and APAH are the most common etiologies, comprising 46% and 51%, respectively, of all PAH cases.^9^ The most frequent causes of APAH include connective tissue disease, human immunodeficiency virus (HIV) infection, portal hypertension, and congenital heart disease.^9^ At the 5th World Symposium on Pulmonary Hypertension in 2013, modifications to Group 1 PAH were proposed, including the removal of persistent PH of the newborn and PH associated with chronic hemolytic anemia due to their now apparent differences when compared with other PAH subgroups. Also, new rare gene mutations, including CAV1 and KCNK3, contributing to HPAH have been identified and thus added to the classification, which includes bone morphogenetic protein receptor-2 (BMPR2) mutations, the most common mutations associated with PAH. Several new drugs have been identified as likely risk factors for drug/ toxin-associated PAH, including interferons, amphetamine-like drugs, and dasatinib (a tyrosine kinase inhibitor).^23^ Accurate classification is important, as it plays an increasing role in directing optimal PAH management.

**TABLE 1:** Updated Clinical Classification of PAH

**Group 1 PH: PAH**	
**Hemodynamic Characteristics**	**Classification**
mPAP ≥ 25 mmHg PAWP ≤ 15 mmHg PVR > 3 Wood units	Idiopathic Heritable BMPR2 mutation Other mutations Drug/toxin-induced Associated with: Connective tissue disease HIV infection Portal hypertension Congenital heart disease Schistosomiasis

*Adapted from Galie N, Humbert M, Vachiery J, et al. 2015 ESC/ERS Guidelines for the diagnosis and treatment of pulmonary hypertension.^12^* *BMPR2 = bone morphogenetic protein receptor-2; HIV = human immunodeficiency virus; mPAP = mean pulmonary arterial pressure; PAH = pulmonary arterial hypertension; PAWP = pulmonary arterial wedge pressure; PH = pulmonary hypertension; PVR = pulmonary vascular resistance.*

### Functional Classification

Functional classification is an important component of the assessment of patients with PAH because it strongly predicts mortality and is an important factor in making decisions about appropriate therapy.^24,25^ The World Health Organization (WHO) FC is the most commonly used tool to assess impact of disease (Table 2).^11^ The New York Heart Association (NYHA) FC, which is similar to the WHO classification, can also be used to characterize patients with PAH and predict outcomes.^26^ In the NIH registry, the median survival time for patients with a baseline classification of NYHA FC I or II was 6 years, compared with only 2.5 years and 6 months for FC III and IV, respectively.^27^

**TABLE 2:** World Health Organization Functional Classification of Patients with Pulmonary Hypertension

**Classification**	**Physical Activity**	**Symptoms** *Dyspnea, fatigue, chest pain, syncope*
Class I	No limitation	None upon ordinary physical activity
Class II	Slight limitation	Symptoms appear upon ordinary physical activity
Class III	Marked limitation	Symptoms appear upon less than ordinary activity
Class IV	Severe limitation	Symptoms appear upon any physical activity or may be present at rest; signs of right heart failure present

*Adapted from Taichman D, Ornelas J, Chung L, et al. Pharmacologic therapy for pulmonary arterial hypertension in adults: CHEST guideline and expert panel report.^11^*

The connection between baseline FC and patient outcomes has been corroborated in other studies, as well. In a study of 162 patients treated with epoprostenol, 3- and 5-year survival rates were 81% and 70%, respectively, for patients with an initial classification of NYHA FC III and only 47% and 27%, respectively, for patients with an initial classification of NYHA FC IV.^28^ Similarly, in another study evaluating PAH patients treated with epoprostenol, 1- and 3-year survival rates were 90% and 71%, respectively, in patients who were classified as FC III at the time of diagnosis, compared with 76% and 47%, respectively, for FC IV patients.^29^ Underscoring the need to improve managed care and clinical approaches to PAH, more than 50% of patients enrolled in the REVEAL Registry and 80% of the patients enrolled in the PHC registry were classified as FC III or higher at the time of diagnosis, indicating that the patients were already experiencing a significant limitation of daily physical activity and had a poorer prognosis than if they had been diagnosed earlier.^7,9^

### Treatment and Management Goals

As PAH therapies have evolved, treatment goals have shifted from short-term functional changes to improvements in long-term outcomes. Current knowledge suggests that composite treatment goals improve outcomes better than any single parameter or tool.^30-32^ On the basis of this perspective, McLaughlin et al. (2009) proposed that PAH management should aim to achieve the following goals, given their prognostic value: modified NYHA FC I or II; normal/near-normal right ventricle size and function on echocardiography or cardiac magnetic resonance imaging; hemodynamic parameters showing normalization of right ventricular function; 6-minute walk distance (6MWD) > 380-440 meters; cardiopulmonary exercise testing, including peak oxygen consumption, > 15 ml/min/kg and ventilatory equivalent for CO2 (EqCO2) < 45 L/min; and normal B-type natriuretic peptide (BNP) levels.^21,26^ The authors suggest that eventually, this multiparameter goal set may be translated to development of a scoring system that can be used to assess response to therapy.^26^

Clinical trials of PAH therapies are also shifting to examine endpoints that reflect long-term outcomes. Historically, the 6MWD test has been an easy and inexpensive way to assess functional status. Because results correlate with hemodynamic parameters and survival, the 6MWD test has been a primary endpoint for many PAH clinical trials. However, the test has limitations, and recent studies question whether differences in 6MWD reflect clinically meaningful changes in quality of life or survival.^26^ Recent clinical trials have shifted toward use of more practical, combined, events-based outcomes such as clinical deterioration (which incorporates decline in 6MWD), initiation of rescue therapy, hospitalization, transplantation, and all-cause death—measures that are clinically relevant and have longer-term implications. For example, in an analysis of REVEAL data, Burger et al. (2014) showed that PAH-related hospitalizations were associated with worse survival at 3 years.^33^

### Prognostic Factors

Many proposed components of composite treatment goals also have prognostic value and may aid in predicting the PAH course. Using REVEAL Registry data, Benza et al. (2012) proposed an algorithm to calculate a risk score to predict survival in PAH patients based on the following parameters: vital signs, WHO Group 1 subgroup, sex, presence of renal insufficiency, NYHA/WHO FC, 6MWD, N-terminal prohormone BNP (NT-proBNP) or BNP level, presence of pericardial effusion, diffusing capacity of lung for carbon monoxide, and hemodynamics (e.g., mean right atrial pressure, and pulmonary vascular resistance). In a cohort of 504 newly diagnosed patients, 1-year survival was accurately predicted using the risk score.^30^ Nickel et al. (2012) systematically evaluated a series of 4 prognostic markers that were independently associated with better survival at baseline and at follow-up (median 38 months), including changes in NYHA FC, NT-proBNP level, cardiac index, and mixed-venous oxygen saturation (SvO2). Analysis demonstrated that NYHA FC I or II, NT-proBNP < 1,800 ng/L, cardiac index ≥ 2.5 L/min/m^2^, and SvO2 ≥ 65, especially at follow-up, were associated with better survival.^31^ Another recent study found that in a univariate analysis, WHO FC III and IV, cardiac index ≤ 2.5 L/min/m^2^, and pericardial effusion at baseline were significantly associated with all-cause death (mean follow-up 46 months). In the multivariate analysis, pericardial effusion, mean right atrial pressure (≥ 10 mm Hg), and low cardiac index predicted higher mortality.^32^

## Guideline-Directed Diagnosis of PAH

As addressed in this section, the guideline-directed diagnosis of PAH involves a thorough patient history and clinical examination to assess symptoms, risk factors, and associated conditions and causes; clinical workup through various imaging, hemodynamic, and cardiopulmonary tests; and the use of biomarkers.

### Review of PAH Pathophysiology

**FIGURE 1:**
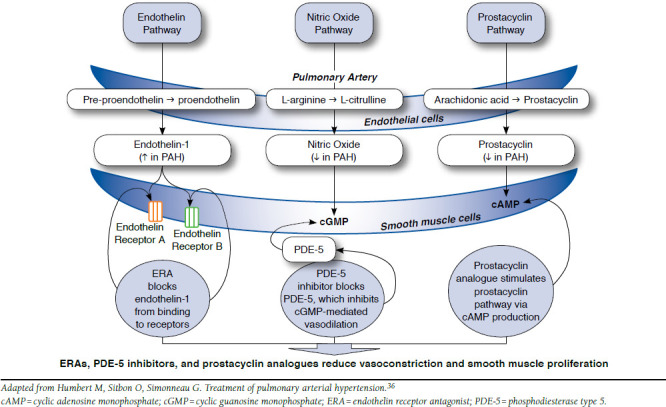
Medical Therapy Targets in Pulmonary Arterial Hypertension

A number of complex molecular and cellular mechanisms contribute to the progressive narrowing of primarily pulmonary arterioles, which leads to increased pulmonary vascular resistance, increased pulmonary arterial pressures, and eventually, right heart failure and death in PAH.^34^ Pulmonary endothelial cell injury is thought to cause dysregulation of molecular signaling pathways, including the nitric oxide, endothelin, and prostacyclin pathways. Downregulation of nitric oxide and prostacyclin production, and upregulation of endothelin production lead to an imbalance of molecular vasoconstrictors and vasodilators (favoring vasoconstriction) and growth factors (favoring remodeling). A pulmonary vasculopathy ensues, characterized by increased vessel tone and thickening of vessel walls as well as in situ thrombosis, which narrows lumen and increases resistance to blood flow (Figure 1).^34-36^ Understanding molecular pathways involved in the pathogenesis of PAH has been key to providing molecular targets for the development of therapies.

### Clinical Presentation of PAH

The evaluation of PAH begins with a thorough history and examination. Patients are often asymptomatic early in the course of their disease or present with nonspecific symptoms, potentially resulting in delayed diagnosis or misdiagnosis. The most common symptoms include dyspnea (83%), fatigue (27%), chest pain (20%), syncope or near-syncope (17%), and cough (14%).^9,21^ In advanced disease, symptoms are related to right ventricular failure, including angina, exertional syncope, peripheral edema, and abdominal distension related to ascites.^37^ Assessment of risk factors and history of associated conditions are also important when considering PAH in the differential diagnosis.^9^

As in symptomatology, early physical exam findings may be subtle but include an accentuated pulmonic component of the second heart sound (S2) and a narrowly split S2. Findings in later disease reflect right ventricular enlargement and failure, as evidenced by the following conditions: right ventricular impulse or heave, a holosystolic murmur of tricuspid regurgitation, third or fourth heart sound, a diastolic murmur that may indicate pulmonic regurgitation, lower extremity edema, jugular venous distension, ascites, hepatomegaly, and pleural effusion.^34,37^ Physical findings suggestive of associated causes of PAH should also be noted. For example, telangiectasia, digital ulceration, and sclerodactyly are seen in scleroderma, while palmar erythema, spider nevi, and jaundice suggest chronic liver disease.^37^

### Workup of PAH

The 2015 ESC/ERS Guidelines for the diagnosis and treatment of PH include an updated algorithm for diagnosis of PAH.^12^ Basic workup of suspected PAH begins with electrocardiogram (ECG) and chest x-ray. ECG may show signs of right ventricular hypertrophy or strain, although sensitivity and specificity of these signs are low. Less commonly, supraventricular tachycardias, including atrial flutter and atrial fibrillation, can be observed in advanced disease. X-ray findings can include central pulmonary arterial prominence, loss of peripheral vascularity, and right atrial and ventricular enlargement.^12,21^

Transthoracic ECG is the best screening test for PAH. Findings suggestive of PAH include right atrial and ventricular enlargement, tricuspid regurgitation, flattening or reverse curvature of the interventricular septum, and underfilled left heart chambers. ECG can also identify congenital defects as a cause of PAH or manifestations of Group 2 to 5 PH. Suggestive findings on ECG should trigger further workup to establish PH and its cause.^12,21^

If screening suggests PH, further testing should start with an attempt to identify more common clinical groups of PH, including chronic thromboembolic PH, left heart disease, obstructive sleep apnea, and other chronic obstructive and restrictive lung diseases. Diagnostics should include ventilation/perfusion scanning, high-resolution computed tomography, polysomnography and oximetry, pulmonary function tests, and arterial blood gases.^12,21^ If testing rules out the more common clinical groups of PH and PAH is suspected, testing to identify potential underlying causes of PAH should be undertaken, including liver function testing and screening for HIV, history of drug/toxin exposure, and connective tissue disease.^12,21^ The 2015 ESC/ ERS guidelines also recommend genetic testing for mutations such as BMPR2 associated with PAH for patients with IPAH or a family history of PAH. The BMPR2 mutation accounts for 75% and 25% of HPAH and IPAH subgroups, respectively.^12^ Patients and their families contemplating genetic testing should consider genetic counseling first.

**FIGURE 2:**
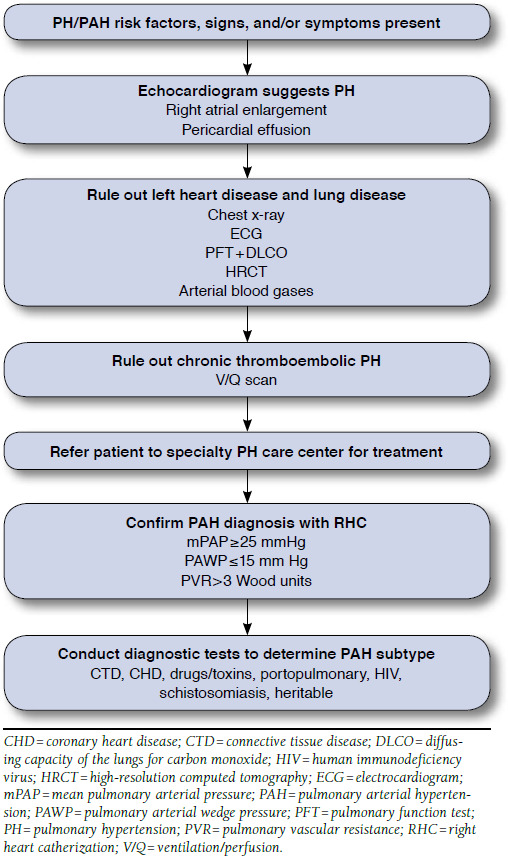
Screening for PAH

RHC, which is required for a definitive diagnosis, defines PAH by specific hemodynamic criteria: mean pulmonary arterial pressure ≥ 25 mm Hg at rest, end expiratory PAWP ≤ 15 mm Hg, and pulmonary vascular resistance > 3 Wood units.^22^ In addition, vasoreactivity testing should be performed at the time of RHC in patients with IPAH, HPAH, or drug/toxin-associated PAH to assess eligibility for CCB therapy.^12^ The newly updated algorithm from the 2015 ESC/ERS guidelines for the diagnosis of PAH is shown in Figure 2.^12^

Recently the use of biomarkers to guide diagnosis and treatment has been an area of active research. For example, elevations of BNP and NT-proBNP correlate with increasing FC and reflect the degree of right ventricular dysfunction and correlate with worse FC, worse pulmonary hemodynamics and mortality, and reductions in response to therapy. Lower baseline and posttreatment levels of BNP are associated with improved survival.^38^ Other potential biomarkers that may be useful tools for predicting prognosis, monitoring disease progression, and assessing response to treatment include troponins, uric acid, d-dimer, and renal function.^38^ Although it is unlikely that one biomarker will replace the need for the current diagnostics, with more research the use of multiple biomarkers could play a larger role in the initial assessment and guide management of PAH.^32^

## Newly Approved and Emerging Therapies and Therapeutic Paradigms in PAH

Recent advances in the medical management of PAH include approvals by the FDA of vascular-targeted therapies that act through different disease pathways and a new therapeutic strategy that involves upfront combination therapy with agents that act through different mechanisms. These advances are reviewed as follows in the context of essential strategies for the therapeutic management of PAH, which involve screening for associated connective tissue disease, stratifying levels of risk, and accounting for supportive or adjunctive therapies.

### Overview of Therapeutic Management of PAH

With increased understanding of the pathophysiology of the disease, the therapeutic management of PAH has evolved greatly over the last 2 decades and now incorporates multiple facets, including prevention, screening, supportive therapies, and targeted drug therapy. Prevention is especially relevant, for example, in cases of PAH related to congenital heart disease, where repair of lesions can eliminate the risk of developing PAH. In addition, decreasing use of medications such as methamphetamine derivatives can prevent drug-related PAH.

A second line of management consists of identifying and screening high-risk individuals based on family history, connective tissue diseases, congenital heart diseases, HIV, and liver diseases. For patients with APAH, which comprises about 50% of all PAH cases, optimizing therapy for their associated condition is a key component of effective management.^9,35^ Screening plays an especially important role in patients whose PAH is associated with connective tissue disease.^32^ The estimated prevalence of PAH in individuals with systemic sclerosis is 10%-12%.^39,40^ Risk factors for development of PAH in these individuals include older age, limited cutaneous subtype, longer disease duration, autoantibody status (positive anticentromere, negative Scl-70), and abnormalities on pulmonary function tests, ECG, and RHC.^39,40^

Given the relatively high prevalence and greater morbidity and mortality associated with PAH in patients who have systemic sclerosis, much work has been done to determine an effective screening strategy. Currently, systemic sclerosis is the only connective tissue disease with recommended screening guidelines. Most algorithms recommend yearly ECG screening in asymptomatic patients with systemic sclerosis or mixed connective tissue disease or other forms with scleroderma features.^40^ The most recently proposed screening and surveillance approach is the 2-step DETECT algorithm.^41^ The first step scores patients using 6 assessments: predicted forced vital capacity divided by predicted diffusing capacity of lung for carbon monoxide; current or past telangiectasias; serum anticentromere antibodies; serum NT-proBNP; serum uric acid; and right axis deviation on ECG. The second step scores ECG findings. Scores from both steps are then combined to determine whether RHC is needed. In a cross-sectional study evaluating this approach, the proportion of missed PAH diagnoses was markedly lower for patients with systemic sclerosis (n = 24) assessed with the DETECT algorithm (4%) compared with the 2009 ESC/ERS screening guidelines (29%).^41^

Risk stratification also plays an important role in the approach to treatment. The 2015 ESC/ERS guidelines recommend risk stratification using a panel of predictive markers. Patients are classified in categories of low, intermediate, or high risk, which predict 1-year mortality (Table 3). Level of risk can then be used to inform treatment decisions. For example, high-risk patients are likely to benefit from more aggressive therapeutic strategies such as combination regimens using intravenous prostacyclins, while low-risk patients can be started on oral therapy.^12^

**TABLE 3:** Pulmonary Arterial Hypertension Risk Assessment

**Prognostic Factors**		**Estimated 1-Year Mortality**		
		**Low Risk (< 5%)**	**Intermediate Risk (5%-10%)**	**High Risk (> 10%)**
Right heart failure		No	No	Yes
Symptom progression		No	Slow	Rapid
Syncope		No	Occasional	Recurrent
WHO FC		I or II	III	IV
6MWD		> 440 m	165-440 m	< 165 m
Cardiopulmonary exercise testing	Peak VO_2_	> 15 mL/min/kg	11-15 mL/min/kg	< 11 mL/min/kg
	VE/VCO_2_ slope	< 36	36-44.9	≥ 45
Serum markers	NT-proBNP	< 300 ng/L	300-1400 ng/L	> 1400 ng/L
	BNP	< 50 ng/L	50-300 ng/L	> 300 ng/L
Echocardiography	RA area	< 18 cm^2^	18-26 cm^2^	> 26 cm^2^
	Pericardial effusion	No	No/minimal	Yes
Hemodynamics	RAP	< 8 mm Hg	8-14 mm Hg	> 14 mm Hg
	CI	≥ 2.5 L/min/m^2^	2.0-2.4 L/min/m^2^	< 2.0 L/min/m^2^
	SvO_2_	> 65%	60%-65%	< 60%

*Adapted from Galie N, Humbert M, Vachiery J, et al. 2015 ESC/ERS Guidelines for the diagnosis and treatment of pulmonary hypertension.^12^* *6MWD = 6-minute walk distance; BNP = brain natriuretic peptide; CI = cardiac index; CMR = cardiac magnetic resonance; FC = functional class; NT-proBNP = N-terminal pro-brain natriuretic peptide; RA = right atrium; RAP = right atrial pressure; SvO_2_ = mixed venous oxygen saturation; VE/VCO_2_ = ventilatory equivalents for carbon dioxide; VO_2_ = oxygen consumption; WHO = World Health Organization.*

Supportive or adjunctive therapies are aimed at managing the sequelae of PAH. Although there are limited data to indicate that these measures have long-term benefits, they can certainly have symptomatic and functional benefits for patients. For example, progression of disease inevitably leads to right heart failure, and resulting fluid overload can be managed with diuretics. In addition, digoxin is sometimes used for inotropic support.^35^ Oxygen supplementation should also be used in hypoxemic patients. Improving arterial oxygenation can significantly improve functional status, and oxygen can also act as a pulmonary vasodilator and thus help decrease pulmonary vascular resistance, although long-term benefit has not been shown.^35,39^ Anticoagulation is also a potential adjunctive therapy, as in situ thrombosis plays a role in the pathophysiology of PAH. However, there are no conclusive randomized data in the peer-reviewed literature. Although some retrospective studies suggest that warfarin may improve survival in some patients, especially in patients with IPAH, HPAH, and anorexigen-APAH, a recent analysis of the REVEAL database showed no survival benefit among patients with IPAH or PAH and connective tissue disease who were treated with warfarin.^42^ Vaccinations to prevent respiratory infections, exercise rehabilitation, and psychosocial care support round out the recommended components of supportive care.^12^

Vascular-targeted therapies are the cornerstone of PAH treatment today. Although there is still no cure for PAH, these treatments have been developed to target the molecular pathways that lead to increased pulmonary vascular resistance, the underlying mechanism of disease.

In cases of failure of pharmacologic treatment, surgical interventions should be considered. Lung transplantation continues to be an option for patients who remain in FC III or IV despite optimal drug treatment. Overall 5-year survival after transplantation has been reported as high as 75% in recent years, and 10-year survival has increased to 45% to 66% with good quality of life.^43^ Thus, early referral for assessment of eligibility for lung transplantation should be advocated.^43^ Another intervention is the percutaneous balloon atrioseptostomy, which creates a puncture in the atrial septum, and septal dilatation, resulting in a right-to-left shunt that unloads the right heart and increases left ventricular preload and output. This procedure can be used in FC IV patients with right heart failure refractory to medical management or with severe syncopal symptoms. Although impact on long-term survival has not yet been demonstrated, this intervention can be used as a palliative measure as well as a bridge for patients awaiting transplantation.^43,44^

### Established Vascular-Targeted Therapies

Four classes of vascular-targeted pharmacotherapies have established prominent roles in the treatment of PAH over the last few decades and significantly improved patient survival. However, these therapies still have limitations, and the disease remains incurable. Introduced in the 1980s, the first agents to gain wide acceptance for PAH treatment were CCBs selected for their vasodilatory effects, such as amlodipine and nifedipine. However, subsequent studies showed that less than 15% of patients with PAH demonstrated vasoreactivity, and only approximately 50% of patients treated with CCBs maintained FC I or II after 1 year of treatment.^45^ Thus, current guidelines recommend that only patients with IPAH, HPAH, or drug-associated PAH who show positive responses after an acute vasodilator challenge during RHC should be treated with CCBs.^11,12,35,46^ A positive vasodilator response is defined by a mean decrease in pulmonary arterial pressure by ≥ 10 mm Hg to an absolute value < 40 mm Hg with unchanged or increased cardiac output.^12^

Owing to downregulation of prostacyclin synthase, prostacyclin levels in PAH are decreased; thus, prostacyclin analogues have been used to stimulate the prostacyclin pathway via cyclic adenosine monophosphate production, resulting in vasodilation and inhibition of smooth muscle growth, and platelet aggregation.^44^ Available prostacyclin analogues can be administered through intravenous (epoprostenol and treprostinil), subcutaneous (treprostinil), inhaled (iloprost and treprostinil), and, most recently, oral (treprostinil) routes. Prostacyclin analogues have been reserved mainly for severe disease (FC III or IV). Significant limitations to their use are related to intravenous administration, which can be complex, requiring substantial patient education and entailing risks of catheter-related infections, venous thrombosis, and rebound PAH.^35,46^ However, intravenous prostacyclin analogues remain an important option for high-risk patients because they improve long-term survival.^43,47^

The endothelin pathway is upregulated in PAH, resulting in vasoconstriction and smooth muscle proliferation. Thus, ERAs have been developed as effective therapies for PAH, demonstrating improvement in exercise capacity, quality of life, hemodynamics, and time to clinical worsening.^35,39^ The 3 currently available oral ERAs are bosentan, ambrisentan, and macitentan. These therapies are generally well tolerated, although bosentan is associated with risks of hepatotoxicity.^39^

PDE-5 inhibitors work on the nitric oxide pathway by blocking the action of PDE-5, which normally inhibits cyclic guanosine monophosphate (cGMP)-mediated vasodilation. The available oral agents are sildenafil and tadalafil, which have been shown to improve symptoms, exercise tolerance, and functional capacity in PAH. They are generally well tolerated but commonly cause headache and flushing.^35,39^

### Newly Approved PAH Therapies

Several new agents have been developed in the last few years and are establishing their role in the management of PAH. Macitentan, the newest ERA, shows increased tissue penetration and greater affinity for and longer duration of blockade of endothelin receptors compared with bosentan.^48^ Macitentan was approved in 2013 after the SERAPHIN trial indicated that its use alone or in combination with other targeted therapies significantly reduced morbidity and mortality in patients with FC II-IV PAH. SERAPHIN was the first event-driven PAH study, using a composite primary endpoint that included worsening of PAH, initiation of prostacyclin analogue infusion, lung transplantation, and death. Macitentan also demonstrated the ability to improve functional status, delay disease progression, and decrease hospitalizations.^49^ This is significant, considering that increased hospitalizations correlate with decreased survival.^33^ Optimal dosing of macitentan is 10 mg orally once daily, and the most commonly observed adverse effects are anemia, nasopharyngitis, bronchitis, headache, influenza, and urinary tract infection. Other concerns are teratogenicity and fluid retention consistent with other agents in this class. Unlike bosentan, macitentan has significantly less hepatotoxicity and does not require mandatory monitoring of liver function.^48,49^

Another new PAH medication is riociguat, a guanylate cyclase stimulator. Upon binding to nitric oxide, guanylate cyclase catalyzes the synthesis of guanosine monophosphate (cGMP), which in turn promotes vasodilation, inhibits smooth muscle remodeling and proliferation, and decreases platelet aggregation. Because nitric oxide is downregulated in PAH, riociguat works by directly stimulating guanylate cyclase independently of nitric oxide.^44^ The PATENT-1 trial showed that riociguat-treated patients had significantly improved 6MWD, the primary endpoint, and also improved secondary endpoints, including pulmonary vascular resistance, NT-proBNP levels, WHO FC, and time to clinical worsening.^50^ PATENT-2, an extension trial of PATENT-1, showed additional increases in 6MWD at 1 year and 2 years of treatment, with > 90% of patients achieving stable or improved FC.^51,52^ Riociguat was FDA-approved in 2013 at a maximum dose of 2.5 mg orally 3 times daily.^48^ Hypotension is a serious adverse effect, and use in combination with PDE-5 inhibitors is contraindicated. Other common adverse effects include headache, dizziness, dyspepsia, peripheral edema, nausea, vomiting, and diarrhea.^48,50^

Prostacyclin analogues have been effective treatments for PAH. However, administration is frequently complicated; thus, these agents are limited to treatment of advanced disease. Treprostinil is the first FDA-approved oral prostacyclin analogue. Its mechanism of action is similar to that of other prostacyclin analogues, promoting vasodilation and inhibiting cell proliferation and platelet aggregation. The FREEDOM-C1 and FREEDOM-C2 studies showed that oral treprostinil (up to 16 mg twice daily) used with background PAH therapy of other classes did not reach statistical significance in the primary endpoint, 6MWD.^53,54^ In the FREEDOM-M trial, treprostinil, when used in treatment-naïve patients as monotherapy at a dose of up to 12 mg twice daily, significantly improved 6MWD; however, no significant improvement in clinical worsening or symptoms was demonstrated.^55^ Adverse effects of treprostinil are headache, nausea, diarrhea, flushing, jaw pain, hypokalemia, and abdominal pain.^53^

Selexipag is a novel agent approved by the FDA in December 2015 to delay disease progression and reduce the risk of hospitalization for PAH. It selectively activates the prostaglandin-I2 receptor agonist, resulting in significant vasodilation.^44^ In the phase 3 GRIPHON study, selexipag at a dose of up to 1.6 mg twice daily reduced the risk of the primary composite endpoint of death or PAH-related complication. The effect was consistent across age, sex, PAH etiology, baseline FC, and background treatment with other PAH medications.^56^ Adverse effects, which were consistent with its mechanism of action, included headache, diarrhea, nausea, jaw pain, myalgias, pain in extremities, flushing, and arthralgias.

### Use of Upfront Combination Therapy

In addition to the development of new agents for PAH, recent studies have indicated that earlier, more aggressive management with upfront combination therapy may benefit patients. This represents a paradigm shift in treatment approach, which has traditionally involved stepwise addition of single agents.^11,12^ In theory, combinations of therapies acting on multiple disease pathways could have synergistic effects and lower dose requirements. A small 2014 pilot study showed that upfront triple combination therapy with intravenous epoprostenol, bosentan, and sildenafil in newly diagnosed FC III or IV patients significantly improved 6MWD and hemodynamics after 4 months of treatment, and at a mean follow-up of 41 months, all 19 patients were still living.^57^

Two larger trials have recently examined the efficacy and safety of combination therapy. COMPASS-2 indicated no significant improvement in time to first morbidity or mortality event in patients who added bosentan to a stable dose of sildenafil.^58^ Conversely, the AMBITION trial established a strong case for upfront combination therapy using ambrisentan and tadalafil compared with pooled monotherapy in FC II or III PAH patients.^59^ The study included 500 treatment-naïve patients who were randomized to combination therapy with tadalafil and ambrisentan or monotherapy with tadalafil or ambrisentan. The study’s primary endpoint was the time to first event of clinical failure, which included death, hospitalization, disease progression, or unsatisfactory long-term clinical response. Mean follow-up period in the study was 517 days. Combination therapy was associated with a significantly lower risk of experiencing the primary endpoint, which occurred in 18% of the combination group, 34% of the ambrisentan monotherapy group, and 28% of the tadalafil monotherapy group (*P* < 0.001). Secondary endpoints at week 24—including 6MWD, percentage of patients with a satisfactory clinical response, and NT-proBNP levels—all improved to a greater extent in patients who received combination therapy. Adverse events, including peripheral edema, headache, nasal congestion, and anemia, occurred more frequently in the combination therapy group than in monotherapy groups, but rates of drug discontinuation were low and serious adverse effects were similar across study groups.^59^

On the basis of the positive results of AMBITION, the FDA recently approved combination ambrisentan and tadalafil for the first-line treatment of PAH.^60^ As discussed by the AMBITION investigators, the study supports a major shift toward early combination therapy that targets multiple disease pathways.^59^ Authors of the 2015 ESC/ERS guidelines for PH cited the AMBITION study results as evidence for their recommended use of combination therapy as a first-line option for patients with PAH in FC II and III.^12^ The 2014 CHEST guidelines for PAH management indicated the potential value of combination therapy strategies for some patients.^11^ Specifically, the guidelines recommend first-line combination therapy with a prostacyclin analogue for high-risk (FC IV) patients.^11^ However, these guidelines were developed and published before the release of the AMBITION trial results; thus, a recommendation for combined ambrisentan and tadalafil as first-line treatment for FC II and III patients was not included.

## Evaluating Current PAH Management Guidelines

Since June 2014, 2 updated sets of evidence-based and expert consensus practice guidelines for PH and PAH have been published. This section summarizes key features of the new guidelines.

### CHEST Guidelines 2014

The CHEST guidelines for PAH, released in June 2014, focus primarily on pharmacologic therapy but do emphasize several key points on diagnosis.^11^ The guideline authors recommend that patients should be evaluated at an expert center prior to treatment when possible to ensure accurate diagnosis of PAH, and ongoing management should involve a collaborative effort between local physicians, specialists, and the expert center. Also, the guidelines reiterate that RHC is required for diagnosis, and vasoreactivity testing should be conducted whenever possible in appropriate patients. Despite these well-established recommendations, a number of studies have shown that adherence to them is low. For example, referral of PAH patients to expert centers is often delayed.^2^ Furthermore, RHC is not always conducted to confirm a PAH diagnosis, and vasoreactivity is not always performed when indicated, often leading to inappropriate use of CCBs.^3^

The 2014 CHEST guidelines offer an approach to managing PAH-specific treatments based on FC. In general, the guidelines advocate stepwise addition of therapies in which patients in FC II or III without evidence of rapid progression or poor prognosis should be treated initially by oral monotherapy with an ERA, a PDE-5 inhibitor, or a guanylate cyclase stimulator. In FC III patients with rapid progression, poor prognostic markers, or progression despite one or more oral therapies, the guidelines recommend initiating an infused prostacyclin. For FC IV patients, infused prostacyclin is the recommended option. Although the 2014 CHEST guidelines authors discuss the prospect of upfront combination therapies, they do not make a recommendation for this approach in patients with low- or intermediate-risk disease. As noted earlier, however, the publication of the 2014 CHEST guidelines predated the release of AMBITION trial results. In addition, selexipag was not included in the CHEST guidelines, as the GRIPHON trial had not yet been completed and the therapy was not yet approved.^11^

### 2015 ESC/ERS Guidelines

The latest set of guidelines on the management of PAH was released by the European Society of Cardiology and the European Respiratory Society in August 2015.^12^ The ESC/ERS diagnostic algorithm begins with screening ECG, with high or intermediate probability PH findings leading to further evaluation to exclude other causes of PH before proceeding with specific tests for various associated causes of PAH. These guidelines also emphasize the need for RHC for definitive diagnosis and the importance of early referral to expert care centers.

**FIGURE 3:**
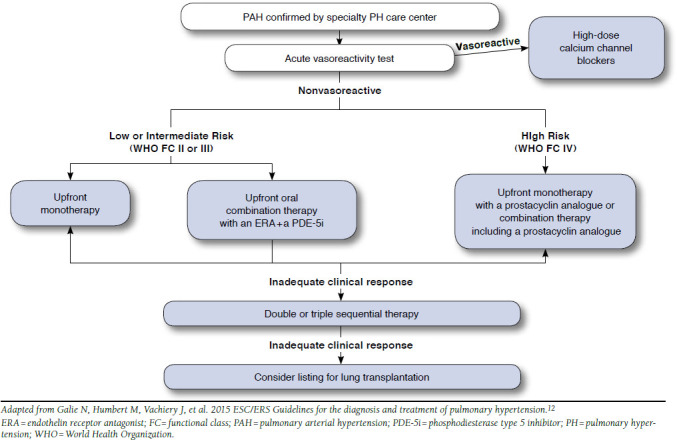
PAH Treatment Algorithm

Compared with the 2014 CHEST guidelines, the 2015 ESC/ ERS guidelines present the stepwise (initial monotherapy) and upfront combination therapy approaches to initial management of FC II to III patients as equal options in the proposed treatment algorithm (Figure 3). The guideline authors give therapy regimens for both strategies a Class I recommendation, indicating that the evidence and/or consensus views support the benefits, usefulness, and effectiveness of initial stepwise and upfront combination approaches. However, it is important to consider that the Class I recommendation for upfront combination therapy applied only to tadalafil and ambrisentan for Class II or III patients, based on the AMBITION trial, and other upfront combinations received weaker recommendations due to the lack of supporting evidence.^12,59^ Also, there have been few direct comparisons between oral monotherapies; thus, ERAs, PDE-5 inhibitors, riociguat, and selexipag all have Class I recommendations as initial monotherapies for FC II and III patients.^12^

The 2015 ESC/ERS guidelines also provide important guidance on use of assessment tools, including symptoms and signs of right heart failure; exercise capacity (e.g., FC, 6MWD, and cardiopulmonary exercise testing); biomarkers (e.g., NT-proBNP, troponins); ECG (e.g., right atrial area and pericardial effusion); and hemodynamics (e.g., mean right arterial pressure, cardiac index, and SvO2) to predict prognosis and mortality risk (Table 3). Furthermore, the 2015 ESC/ERS authors advocate for goal-oriented therapy (vs. nonstructured approaches) by using these risk classifications to assess treatment response. The main treatment goal is to achieve or maintain a “low risk” profile (e.g., good exercise capacity, intact right ventricular function, and no right heart failure).^12^ The advantage of this strategy is that even patients who improve or stabilize on initial therapy can still receive additional therapy if goals are not met. Improvements and deteriorations in these parameters after initiation of PAH-targeted therapy have a high predictive value and are strongly associated with survival outcomes.^31^

## Identification of Gaps for Future Study and Guideline Development

Although both sets of recently published guidelines reflect the significant developments that have emerged in PAH diagnosis and therapy, they also highlight the gaps that remain in the understanding of the disease and its optimal management.^61^ The limitations of these guidelines reflect the paucity of high-quality clinical trials on PAH therapies. Most recommendations in the guidelines were not supported by high-level evidence and rather, were primarily consensus-based recommendations. For example, the guidelines are unable to recommend one agent or class of agents, supporting the need for more research on comparative effectiveness of available drugs using clinically relevant and event-driven endpoints. There is also the question of whether efficacy may differ among patient subgroups. Furthermore, perhaps the next guidelines will be able to more definitively provide guidance on whether sequential combination of therapies or upfront combination therapies are more beneficial. No studies thus far have compared these 2 strategies directly, so both are recommended as options in low- and intermediate-risk patients in the 2015 ESC/ERS treatment algorithm. Better understanding of which low-/intermediaterisk patients may benefit from combination therapy versus monotherapy can also help manage costs, eliminating the need for additional agents in patients who may achieve similar outcomes on initial monotherapy and still have the opportunity to receive additional agents sequentially when treatment goals are not met.

The current guidelines also underscore the issues that are still unaddressed in PAH management. For example, there is little guidance on the management of FC I patients other than the “watchful waiting” approach. This highlights the need to better understand and identify markers of progressive disease to help determine when and how to treat at-risk patients.

## Managed Care Strategies for Improving Outcomes in PAH

PAH remains a progressive disease that carries high morbidity and mortality for patients and engenders a significant economic burden. Thus, optimizing management of patients with PAH is critical. Barriers remain, however, and include factors such as evolving understanding of the disease, need to increase clinician awareness and continuing education on PAH, health insurance coverage and prior authorization requirements, and need for additional quality clinical trials to guide various aspects of PAH management.

Currently, guidelines recommend early referral to expert centers to ensure timely and accurate diagnosis and initiation of appropriate treatments.^11,12^ This guidance is supported by observations that survival of patients with PAH is significantly improved at referral centers, while delayed referral can adversely affect prognosis.^2,62^ According to the 2015 ESC/ERS guidelines, an expert center’s role is to assess and investigate all causes of PH, manage PAH-specific therapies, and work collaboratively with other health care providers, including primary care, to optimize patient outcomes. Centers should have a multidisciplinary team that includes physicians, pharmacists, and radiologists, as well as psychological and social work support and facilities that can provide intensive therapy, range of diagnostics, and emergency care.^12^

However, despite educational initiatives, delayed referral of patients to expert centers continues to be problematic. Some patients are referred with incomplete diagnostic testing or misdiagnoses as well as inappropriate treatments. Still others are not referred until late in the course of their disease.^2^ As a result, these patients may have missed the opportunity to gain any survival benefit associated with early treatment. Also, patients with later-stage disease require more intensive treatments and likely have increased frequency and duration of hospitalizations, adding to the cost burden of PAH. Thus, establishing programs and further education initiatives to ensure early referral to expert centers may help contain high costs and resource utilization associated with PAH.

Many of the strategies proposed to improve outcomes and decrease costs of PAH rely heavily on the referral of patients to tertiary care centers. However, expert center referral rate is often low and delayed. Reasons for this have not been formally studied, but possible explanations include the increasing decentralization of PH care, with local specialists managing more patients on their own partly because of increased awareness of the disease and the increased availability of easily prescribed (but not necessarily easily managed) oral therapies.^63^ Also, patients may be reluctant to visit PH centers if significant travel to an unfamiliar urban area is involved. Thus, patients may be receiving care from an increased number of providers with a wide range of expertise, leading to nonuniform care.

On the basis of these trends, the Pulmonary Hypertension Association has shifted its approach from growing the PH community to “establish[ing] a program of accredited centers with expertise in pulmonary hypertension that aspires to improve overall quality of care and ultimately improve outcomes of patients with pulmonary hypertension.”^63^ The Pulmonary Hypertension Care Center committee was formed with a goal to raise the level of care in all centers by creating an accreditation program that promotes standards and adherence to published guidelines and consensus statements as well as fostering collaboration among expert centers for patient management and research.^63^ These initiatives would potentially significantly improve patient referrals to expert care and contribute to improved outcomes as well as cost containment in the PAH patient population. With such referrals, it may be helpful to create comanagement arrangements that may help alleviate concerns about “stealing” patients.

Another strategy that may contribute to improved outcomes is the establishment of additional screening guidelines for various at-risk groups, although further understanding of the pathogenesis of individual subgroups may be needed. Effective screening can help ensure early diagnosis and thus initiation of treatment. Studies have already established that early treatment of PAH is beneficial.^64^ In the well-studied case of PAH in patients with systemic sclerosis, the poor prognosis in these patients may be partially due to delayed diagnosis, and screening programs have improved survival rates by ensuring early diagnosis and initiation of treatment.^65^ If similar strategies can be applied to other at-risk groups as well as patients with PAH FC I, similar benefits may be seen yielding improved clinical outcomes and delayed progression of disease.

Clearly, education for health care providers continues to be a need, given the many recent developments in new treatment options and evolving understanding of best approaches to PAH management. Practice trends show that there is under-utilization of treatment guidelines, and patients are generally undertreated, especially in late disease, and often treated with inappropriate medication options.^2-4^ While early referral to expert centers should still be advocated as a primary strategy to ensure initiation of early and optimal treatment, there is still a need to educate local primary care and specialist physicians who should be equipped to work collaboratively with expert center providers. Some strategies that have been proposed include programs that generate automated reminders at follow-up and implementation of standing orders to improve compliance with guideline-recommended diagnostics and therapies.^3^

The optimal treatment strategy for PAH patients is still an area of research, and further investigation addressing various aspects of PAH management will undoubtedly improve patient outcomes and decrease the economic burden of PAH. Sikirica et al. (2014) analyzed health care costs for PAH patients before (baseline) and after (follow-up) initiation of PAH medications and showed that although pharmacy costs increased significantly in the follow-up period, these were offset by significantly lower medical costs due to fewer outpatient and inpatient encounters.^15^ However, the study also indicated that more than 50% of subjects discontinued, switched, or augmented their initial PAH medication. The investigators suggest that further research may be needed to examine treatment patterns among patients in various subgroups of PAH (e.g., age, sex, PAH subtype) to determine whether these are clinically optimal and cost-effective strategies.^15^

Supportive care should not be overlooked for patients with PAH, many of whom have comorbid conditions that contribute to high cost and resource utilization. Analyses of managed care databases observe that PAH patients have significantly higher baseline comorbidities such as hypertension, diabetes, and congestive heart failure. They also have increased PAH-associated comorbidities such as connective tissue disorders and respiratory infections.^14,15^ These are reminders that optimal management of underlying disorders and preventive measures such as vaccinations are also important in cost containment in the PAH population.

The recent evidence from the AMBITION trial raises the question of whether more aggressive initial upfront combination therapy may be superior to a sequential approach to treatment of PAH. However, the additional costs and resource utilization associated with combination therapy, such as potentially greater need for monitoring and management of adverse effects, are issues that pose challenges for managed care professionals and providers. Future cost and comparative effectiveness studies and additional clinical trials will be essential for guiding strategies to address these challenges and ensure that patients with PAH receive appropriate therapies.^66^

## Summary and Conclusions

Recent advances in the field of PAH offer considerable promise for closing documented gaps in diagnostic processes, treatment, and ongoing patient management. As reviewed in this article, especially noteworthy advances include deeper insights into the pathogenic mechanisms of PAH, the availability of new therapies that improve patient function and reduce risks of morbidity and mortality, clinically relevant approaches to conducting clinical trials on PAH therapies, and evolving evidence-based guidelines that include new recommendations for therapeutic management strategies, including upfront combination therapy. To realize the potential for improving patient outcomes and achieving treatment goals through these advances, managed care professionals and providers face important challenges in promoting awareness of successful methods for recognizing PAH symptoms, conducting accurate differential diagnoses, referring patients to centers of expertise, and making effective decisions to ensure that patients receive appropriate therapies according to evidence-based and expert consensus guidelines. In these efforts, coordinated and collaborative approaches to care—involving primary care providers, specialists in pulmonology and cardiology, pharmacists, and managed care decision makers—will be essential.

## Expert Commentary

### Nicholas S. Hill, MD

The past few years have been a very exciting time in the field of pulmonary hypertension. We have witnessed the successful performance of longer-term event-driven trials, the introduction of 4 new pharmacotherapies to the market, and the completion of the first large-scale trial of upfront combination therapy, yielding highly favorable results. The field has moved so rapidly that guidelines formulated more than a year ago are now out of date. Yet we still have many major challenges. We still diagnose patients fairly late in the course of their disease. This is related to many factors, including the insidious rate of progression we see in some patients, delays on the part of patients in seeking medical attention, misdiagnoses, and delays in recognition on the part of health care providers. Patients still undergo incomplete evaluations that lead to inappropriate treatment regimens. Also, despite the recent advances in therapeutics, the average patient gets a partial response, and although the indications are that we have made significant gains in patient survival, we are nowhere near a cure.

This article on developments in the field of PAH aims to bring us up-to-date on the recent advances. It examines the problems with early recognition and diagnosis of the disease and reviews current standards on the diagnostic approach. It underlines the importance of adhering to guidelines, the essential role of right heart catheterization in characterizing and confirming the disease, and the advisability of referral to expert pulmonary hypertension centers to ensure appropriate evaluation and treatment. The article also reviews the current status of pharmacotherapy, including a more detailed consideration of the newer agents and the upfront combination therapy examined in the AMBITION trial. The most recent guidelines are compared and contrasted, including those from the American College of Chest Physicians as well as from the European Society of Cardiology and the European Respiratory Society. Additionally, the article raises concerns about the cost-effectiveness of some of our current therapeutic approaches. After reading the article, managed care professionals and providers will have an up-to-date perspective on PAH management and an appreciation of the gains we have made as well as the persisting gaps in care.

### Michael J. Cawley, PharmD, RRT, CPFT, FCCM

PAH continues to present challenges to managed care professionals and providers in all aspects of care that include early and accurate diagnosis, prompt referral of patients to centers of expertise, identification of appropriate pharmacological therapies, and overcoming of barriers to therapy access. Working in collaboration with PAH expert centers, health care providers and managed care professionals can become better educated to help identify patients at risk so that early diagnostic and therapeutic interventional measures can be implemented to prevent disease progression.

The clinical practice guidelines from the 2014 CHEST and the 2015 ESC/ERS statements provide the best evidence to date for determining optimal drug selection for patients with PAH. Although both sets of guidelines provide an exhaustive review of the evidence, the quality of evidence is limited, with many of the recommendations based on expert opinion. The authors of this manuscript agree that the current best-practice evidence supports the use of monotherapy or oral combination therapy in patients with WHO FC II-III and initial combination therapy in WHO FC IV. In addition, we concur that combination therapy may also be added to WHO FC II and III patients, based on recent published evidence. Clinical trial endpoints have been traditionally targeted on measuring a variety of short-term symptomatic and functional benefits to patients. Inconsistency in clinical trial endpoints has limited the ability of clinicians to come to an overall consensus in drug therapy selection. However, more recent data has provided very strong evidence that more aggressive upfront combination therapy has shown significant improvement in more long-term benefits, including morbidity and mortality. This approach from stepwise treatment to upfront combination therapy may have advantages. Combination therapy may indeed impact multiple biological and molecular pathways and could have a synergistic effect, making its efficacy more pronounced with the potential of lower serum drug concentrations. Many issues still need to be addressed in future clinical studies to better assist clinicians in optimal selection of drug therapy options. These issues include direct comparisons between oral therapies, comparative effectiveness of different combined agents, sequential monotherapy versus upfront combination therapy, and appropriate therapy regimens for patients classified by low, medium, and high risk.

In addition to assessing the effects of PAH therapies on patient-centered outcomes, including quality of life and function, new studies are needed to address economic issues associated with the disease. These areas of research may include cost-avoidance studies in patients identified early in the disease and comparisons of costs associated with adverse effects in patients receiving upfront combination therapy versus monotherapy. In addition, future research may address the influence of PAH therapeutic interventions on preventing hospital readmissions to address the Centers for Medicare & Medicaid Services’ Hospital Readmissions Reduction Program, which reduces payments to hospitals with excessive readmissions. Finally, less invasive screening methods and technological advances in point-ofcare prognostic, biomarker, and genetic testing could expedite patient referral, diagnosis, and therapeutic intervention.
